# Radial neuropathy due to occupational lead exposure: Phenotypic and electrophysiological characteristics of five patients

**DOI:** 10.4103/0972-2327.53080

**Published:** 2009

**Authors:** N. Shobha, Arun B. Taly, Sanjib Sinha, T. Venkatesh

**Affiliations:** Department of Neurology, National Institute of Mental Health and Neurosciences (NIMHANS), India; 1 Department of Biochemistry, St John's Medical College and Hospital, Bangalore, India

**Keywords:** Electrophysiology, lead, neuropathy, radial nerve

## Abstract

Lead is a ubiquitous and versatile metal that has been used by mankind for many years. It is a toxic heavy metal that ranks as one of the most important environmental poisons in the world. Research conducted in recent years has increased public health concern about the toxicity of lead at low doses and has supported a reappraisal of the levels of lead exposure that may be safely tolerated in the workplace. Neuropathy is one complication of lead poisoning. The aim of this study is to describe the phenotypic and electrophysiological profile in five male patients working in a battery factory who developed radial nerve neuropathy due to lead exposure. All patients had elevated blood lead levels that were in the toxic range. The concerned regulatory bodies should make it mandatory for workers to undergo regular health checkups to detect signs of lead poisoning and must ensure that workers are aware about the ill effects of exposure to this metal. Chelation therapy removes lead from the blood and soft tissues and chronic lead exposure often requires repeated courses of treatment.

## Introduction

The common causes of neuropathy in India include Hansen's disease, diabetes mellitus, Guillain-Barré syndrome, chronic inflammatory demyelinating neuropathy, genetically-determined neuropathy, and various drugs.[1] Exposure to toxins like acrylamide, carbon disulfide, inorganic mercury, methyl n-butyl ketone, polychlorinated biphenyl, thallium, triorthocresyl phosphate, arsenic, lead, styrene, and toluene could result in neuropathy, and correct diagnosis calls for a high index of suspicion.[2]

Patients with lead neuropathy present with weakness that primarily involves the wrist and finger extensors, but which could also spread to involve other muscles.[3] Patients with unusually long exposure to inorganic lead may show mild sensory and autonomic neuropathic features rather than the motor neuropathy classically attributed to lead toxicity.[4] Lead intoxication in humans causes axonal degeneration, but in some other species it causes a primarily demyelinating neuropathy. Diagnosis of lead neuropathy is important because it is potentially reversible and also because its early detection and treatment may prevent other systemic complications. There are only a few reports of lead neuropathy from India.[5]

The aim of this study is to describe the phenotypic and electrophysiological profile of five patients with lead neuropathy.

## Materials and Methods

In this retrospective audit, we reviewed the case records of five patients who had been diagnosed with lead neuropathy at the Department of Neurology, National Institute of Mental Health and Neurosciences (NIMHANS), Bangalore, India, during the period from 1992 to 2006. The clinical diagnosis of lead neuropathy was based upon the finding of asymmetric or symmetric distal motor weakness, without sensory symptoms or signs, in the presence of a history suggestive of lead exposure. The diagnosis of lead neuropathy was confirmed by the presence of elevated blood lead levels (considered acceptable up to 9 μg/dl). For measuring blood levels we used the ESA lead analyzer in the Department of Biochemistry, St. John's Medical College and Hospital, Bangalore, India.

Detailed clinical and occupational history, demographic data, observations made during systemic evaluation (especially with regard to anemia, nails, gums, etc.), and results of standard neurological examination (including cognition, motor power, tendon reflexes, and sensory examination) were recorded. Investigations done included routine urine analysis, complete hemogram, and serum biochemistry, including measurement of blood levels of lead. Bone marrow and iron studies were not carried out to exclude other causes of anemia. Motor (median, ulnar, and radial nerves in the upper limbs and common peroneal and posterior tibial nerves in the lower limbs) and sensory (median, ulnar, and radial nerves in the upper limb and the sural nerve in the lower limb) nerve conduction studies were carried out in all the patients. The following parameters were recorded: distal latency, amplitude of compound motor action potential (CMAP) and sensory nerve action potential (SNAP), conduction velocity, and F wave persistence and latency. All the data were entered in Microsoft Excel for analysis.

## Results

Five men were diagnosed to have lead neuropathy. Their ages ranged from 30 to 37 years. There was history of occupational exposure to lead in all cases, i.e., they had been working in battery shops or manufacturing units for a mean duration of 7 years. All of them presented with history of wrist-drop and finger drop. None of the patients had sensory symptoms. The demographic and clinical features are shown in the Table 1. We describe one of these patients who presented with the classical features of lead neuropathy.

**Table 1:** Demographic, clinical, and laboratory features of patients with lead neuropathy

Age (years)	Occupation	Duration (years)	Presenting symptom	Onset and duration of neurological manifestation	Systemic symptoms	Systemic signs	Hb (gm/dl)	Blood lead level (μg/dl)
30	Battery factory	15	Right and left wrist-drop	Acute onset; 15 days	Pain abdomen - 1 year	Pallor, lead line on gum	9.0	37.8
37	Battery factory	10	Right and left wrist-drop	Acute onset; 6 months	Constipation - 2 years, pain abdomen - 1 year, sweating forehead - 6 months	Pallor, lead line on gum	9.2	46.0
35	Battery shop	Not available	Right and left wrist-drop	Insidious onset; 2 months	Nil	Pallor, lead line on gum	11.4	55.8
30	Battery factory	15	Bilateral wrist-drop R > L	Insidious onset; 3 months	Nil	Pallor	10.5	107.8
32	Battery factory	7	Weakness of right index, middle, and ring fingers	Insidious onset; 3 months	Nil	Nil	14.4	50.5

**Figure 1:**
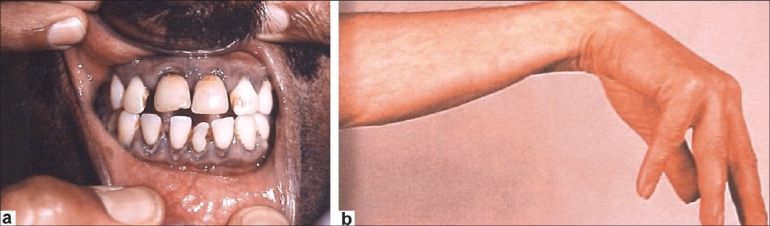
(a) Patient 2 with lead line in the gingiva; (b) wrist-drop in patient 3

Except for patient 5, all others had anemia. None of our patients had basophilic stippling in the peripheral blood smear. The blood lead level was elevated 4-12 times above normal. Patients 1 and 2 had lead lines in the gum [Figure 1a]. Urinary porphobilinogen was negative. Due to nonavailability of the required facilities at our center, serum porphyrin level was not analyzed.

### Patient 3

A 35-year-old gentleman, presented with insidious-onset weakness of the right wrist of 2 months' duration; the weakness of the right wrist had been followed, 15 days later, by similar weakness of the left wrist. There were no sensory symptoms. He had been employed in a battery shop for the last 8 years. On examination, there was pallor and a dark line on the gums. Neurological examination revealed wasting and weakness of the wrist extensors bilaterally (MRC grade: 3/5) [Figure 1b]. There was minimal reduction in pain sensation along the ulnar border of the right forearm. The blood lead level was 55.8 μg/dl (acceptable limit: 9 μg/dl). The peripheral smear did not reveal basophilic stippling. A diagnosis of lead neuropathy was considered and Penicillamine 250 mg twice a day was initiated. After 25 days, there was an improvement of 25% in power, and at the follow-up visit after 4 months his power had improved by 50%.

The peripheral nerve conduction parameters are mentioned in [Table 2]. The conduction velocities were normal in all patients. In addition, patient 1 had reduced CMAP amplitude of the right common peroneal and patients 3 and 5 had reduced CMAP amplitude of the right median nerve. Most of the sensory conduction parameters were normal in all the patients, except for slightly decreased SNAP of the right radial nerve in patient 3 and mildly decreased SNAP of the right median and ulnar nerves in patient 4, suggesting mild sensory involvement.

**Table 2:** Electrophysiological parameters in patients with lead neuropathy

Nerves	Parameters	Controls	Patient 1		Patient 2		Patient 3		Patient 4		Patient 5	
							
			Right	Left	Right	Left	Right	Left	Right	Left	Right	Left
**Motor**												
Median	DL (ms)	2.6 ± 0.5	4.0	-	4.3	4.0	3.5	4.7	4.2	3.9	3.3	5.5
	CMAP (mV)	14.5 ± 3.4	9.9	-	6.5	9.1	5.6	11.7	10.5	12.1	6.5	9.5
	CV (m/s)	58.7 ± 3.8	46.9	-	54.9	53.8	51.3	40.4	51.2	50.0	57.1	59.0
	F Persistence	-	16/16	-	4/16	9/16	12/16	16/16	16/16	15/16	-	-
	F Lat (ms)	25.0 ± 1.8	31.1	-	28.1	28.0	27.7	31.2	26.5	28.1	-	-
Ulnar	DL (ms)	2.0 ± 0.5	3.4	-	3.0	2.7	3.5	3.3	3.2	3.2	2.7	2.2
	CMAP (mV)	10.9 ± 2.6	7.9	-	7.5	6.9	9.4	11.4	10.7	6.9	5.1	4.9
	CV (m/s)	59.9 ± 4.8	41.2	-	52.2	57.5	51.4	40.7	56.1	45.0	55.8	67.6
	F Persistence	-	16	-	9/16	11/16	14/16	16/16	13/16	16/16	12/16	-
	F Lat (ms)	25.7 ± 1.9	31.3	-	28.0	26.8	28.8	28.3	25.3	28.3	28.6	-
Radial	DL (ms)	-	3.0	-	Absent response	3.9	3.0	6.6	4.0	4.3	3.3	3.0
	CMAP (mV)	-	1.09	-		0.16	3.1	2.5	2.3	2.4	1.9	3.6
	CV (m/s)	-	42.9	-		50.0	40.0	52.9	42.5	48.3	67.8	66.7
	F Persistence	-	Absent	-		Absent	Absent	Absent	Absent	16/16	Absent	-
	F Lat (ms)	-		-						31.3		-
CP	DL (ms)	3.6 ± 0.5	6.4	-	5.5	-	4.5	5.2	5.1	5.4	3.8	6.0
	CMAP (mV)	8.0 ± 2.9	3.2	-	10.4	-	8.8	5.3	7.6	6.6	10.7	11.9
	CV (m/s)	51.1 ± 5.5	37.3	-	47.5	-	54.5	51.0	57.4	57.4	51.6	50.0
	F Persistence	-	6/16	-	4/16	-	16/16	14/16	3/16	5/16	-	-
	F Lat (ms)	42.8 ± 3.1	54.3	-	48.3	-	37.6	45.1	34.3	32.3	-	-
**Sensory**												
Median	DL (ms)	-	2.5	-	2.6	2.1	2.4	3.1	3.3	3.08	2.9	2.4
	SNAP (μV)	29.9 ± 8.1	30	-	13.7	10.3	23.0	14.0	9.0	14.1	18.2	23.4
	CV (m/s)	59.5 ± 4.2	54.7	-	56.8	57.9	58.3	41.7	48.2	51.9	58.2	62.5
Ulnar	DL (ms)		2.7	-	2.08	2.5	3.0	2.6	3.7	2.7	2.5	2.8
	SNAP (μV)	22.5 ± 6.7	18.3	-	10.6	12.9	10.7	18.6	7.4	16.3	16.1	8.8
	CV (m/s)	56.2 ± 4.1	43.5	-	57.8	63.5	40.0	43.6	37.6	44.2	55.6	59.0
Radial	DL (ms)	-	2.6	-	2.0	18.8	2.4	2.7	2.1	2.4	1.9	2.08
	SNAP (μV)	-	17.2	-	29.1	23.0	6.8	25.8	24.6	21.9	49.4	22.0
	CV (m/s)	-	53.8	-	67.5	69.1	57.4	51.5	66.0	59.3	71.4	67.3
Sural	DL (ms)	-	3.2	-	2.2	-	3.1	5.6	2.7	2.7	2.6	3.04
	SNAP (μV)	21.5 ± 8.5	13.0	-	21.1	-	14.0	24.4	11.1	11.6	13.5	15.4
	CV (m/s)	48.4 ± 4.7	43.8	-	57.9	-	44.3	25.2	51.5	51.3	53.0	46.1

DL: Distal latency; CMAP: Compound action muscle potential; CV: Conduction velocity; SNAP: Sensory nerve action potential; ‘-’: Not done/available

Three of the patients[1,3,5] were treated with oral d-penicillamine (500–750 mg/day), while patients 2 and 4 received meso 2,3 dimercaptosuccinic acid (DMSA). Follow-up data was available only for patient 3.

## Discussion

Lead is used in industries such as construction, ceramics, paints, plastics, and metallurgy and hence lead poisoning can be considered a common occupational hazard. Inhalation of inorganic lead in the form of fumes, vapors, and mists is a major form of exposure in the occupational setting. Environmental exposure to toxic lead levels due to soil, food, and water contamination can also occur. The common sources of lead poisoning are fumes from burnt car batteries, ingestion of flaking paint, inhalation of vehicle fumes, consumption of food cooked in cheap aluminum or brass utensils or in ‘kalai,’ i.e., vessels that are poorly coated with tin adulterated with lead, and application of ‘kajal’ (mittal). Tesink *et al*. reported that poisoning is almost always caused by ingestion. Lead is absorbed from the respiratory tract into the circulation and is transported on the surface of the red cell, which carries most of the absorbed lead.[6] There are three compartments in the body where lead can be stored: the RBC pool, the soft tissues, and the skeletal system (stores 95% of the body lead). Though the kidneys excrete lead, only a small proportion of the total body lead is removed, and continued exposure results in accumulation of the metal in the tissues. Some people, perhaps due to genetic factors, are more susceptible to poisoning than others. About 15% of Caucasians have a variant of a gene which encodes for aminolevulinic acid dehydrogenase, a critical enzyme in the production of haem, which may make them more susceptible to toxicity from retained lead. Low levels of calcium, iron, copper, zinc, or phosphorus in the diet or high levels of fat can increase lead absorption.[1]

A classical description of lead poisoning has been provided by Van Gogh in his autobiographical letters. The symptoms include initial debilitation; stomatitis, with loss of teeth; recurring abdominal pains; anemia (with a ‘plumbic’ skin tone); neuropathy of the radial nerve; and a saturnine encephalopathy, with features such as epileptic crises, progressive changes in character and periods of delirium.[7] Lead poisoning commonly manifests with unexplained abdominal colic, constipation, wrist-drop, footdrop, anemia, hyperuricemia, and hepatosteatosis. Secondary hypertension following excess exposure to lead has been observed due to its effect on the myocardium and the renal circulation.[8] Cognitive decline has been reported following chronic lead exposure.[9] Chronic intoxication with lead and sulfur compounds may rarely cause Parkinson's disease.[10] Neonates may present with encephalopathy and seizures.[11]

A typical patient of lead neuropathy presents with symmetric distal upper limb weakness and wasting, especially of the forearm and hand muscles; the extensors of the wrist may be selectively weak. The differential diagnosis includes multifocal motor neuropathy, inclusion-body myositis, specific compressive mononeuropathies, posterior interosseous syndrome, focal forms of motor neuron disease, and hereditary predisposition to pressure palsy. There is axonal type of involvement of the peripheral nerves in the upper limbs. The neurologic manifestations of lead depend on the duration of exposure, in that a shorter duration may predispose them to motor neuropathy, as in our series. Rubens *et al*. (2001) reported 46 patients with neuropathic features who had been exposed to lead for periods ranging from 8 to 47 years (mean 21.7 years). All of them showed mild sensory and autonomic neuropathic features rather than just the motor neuropathy that is classically attributed to lead toxicity. All of them had distal paraesthesiae, pain, impaired pin-prick sensation, diminished or absent ankle jerks, and autonomic vasomotor or sudomotor disturbances. Reduced vibration sensation and postural hypotension were present in all the 20 patients studied. Motor conduction velocity and CMAP amplitudes were normal, with marginally prolonged distal motor latencies. SNAP amplitudes lay at the lower end of the normal range, and the distal sensory latencies were prolonged.[4] The patients in this study had a predominantly axonal type of neuropathy.

Neuropathic features develop only when lead levels are more than 70 μg/dl. However, except in one of our patient (patient 4), blood lead levels were less than 70 μg/dl. Chuang *et al.* observed that measurement of vibration sensory threshold is a relatively effective tool for detecting lead neuropathy in field studies and that lead might cause sensory neuropathy with an effect threshold corresponding to a 5-years' mean blood lead concentration of 31 μg/dl. The motor neuropathy associated with subacute poisoning is more likely to be a form of lead-induced porphyria rather than its direct neurotoxic effect. It is proposed that the development of lead neuropathy may depend on inherent factors (like genetic constitution) which determine how it is metabolized in the body. The normal conduction velocity observed in lead neuropathy is in full agreement with the hypothesis that the axonal degeneration is due to the biochemical damage to the perikarya of the anterior horn cells.

Seppalainen *et al*. reported that even in neurologically symptom-free lead workers, motor and sensory conduction velocities are slow and, in addition, electromyographic abnormalities like denervation activity and loss or changes in the motor unit potentials may appear.[13,14] There are contradictory reports on correlation of lead levels with type of peripheral nerve pathology. In one study on lead neuropathy, blood levels of lead were low with demyelinating type of neuropathy and high with axonal type of neuropathy.[15] In another study there was no correlation between the blood lead level and the neurophysiological measurements.[16] Electromyographic abnormalities consist of fibrillations, diminution of the number of motor units on maximal contraction, and an abnormally long duration of the motor unit potentials. Lead may affect central, peripheral, and autonomic nervous systems as shown by prolonged evoked potentials, decreased conduction velocities, and reduced R-R interval variability.[17] These tests however were not carried out in our study.

All but one of our patients had anemia; this anemia may have been secondary to the lead toxicity or, on the other hand, it could be that the anemia made them more prone to lead toxicity. Recurrent abdominal pain or constipation is common in lead poisoning. There are instances of patients having undergone surgeries for the abdominal pain.[18] It was earlier believed that pain abdomen was present in all cases and that without this symptom the diagnosis of lead poisoning could not be made. However, only two of our patients had history of chronic abdominal pain, and only one had constipation. The peripheral blood smear may show anisocytosis, basophilic stippling, and Cabot ring bodies in patients with lead poisoning, but none of our patients had these features.

All the patients in our series were ignorant of the ill effects of lead and took no precautions at work to prevent exposure. On exposure at the workplace, management includes removal of the patient from the source of exposure and chelation therapy with EDTA (ethylene diamino tetraacetic acid), BAL (British antilewisite), dimercaptosuccinic acid (DMSA), or D-penicillamine. These compounds attach to lead and are excreted through the renal route. Oral supplementation with calcium, iron, and thiamine has also been tried as part of the treatment. EDTA, penicillamine, and British antilewisite may decrease blood lead levels but may not improve neuropathy. The prognosis depends on the duration and level of exposure. We had follow-up details for one patient and this patient improved by 50% at the end of 4 months. In the series by Ruben *et al.* (2001), examination of 151 individuals working with lead showed that 46 of them were affected.[4] This has important public health implications. People working with lead must undergo periodic health checkups. However, employers may not cooperate for the fear of losing staff and of the possibility of having to pay compensation; employees, on their part may not cooperate for fear of losing their job. Hence, it may be necessary for the government to enact laws and enforce compliance.
